# Metadynamics Simulation Study on the Conformational Transformation of HhaI Methyltransferase: An Induced-Fit Base-Flipping Hypothesis

**DOI:** 10.1155/2014/304563

**Published:** 2014-06-19

**Authors:** Lu Jin, Fei Ye, Dan Zhao, Shijie Chen, Kongkai Zhu, Mingyue Zheng, Ren-Wang Jiang, Hualiang Jiang, Cheng Luo

**Affiliations:** ^1^College of Life Sciences, Zhejiang Sci-Tech University, Hangzhou 310018, China; ^2^Institute of Traditional Chinese Medicine and Natural Products, Jinan University, Guangzhou 510632, China; ^3^Drug Design and Discovery Center, State Key Laboratory of Drug Research, Shanghai Institute of Materia Medica, Chinese Academy of Sciences, Shanghai 201203, China

## Abstract

DNA methyltransferases play crucial roles in establishing and maintenance of DNA methylation, which is an important epigenetic mark. Flipping the target cytosine out of the DNA helical stack and into the active site of protein provides DNA methyltransferases with an opportunity to access and modify the genetic information hidden in DNA. To investigate the conversion process of base flipping in the HhaI methyltransferase (M.HhaI), we performed different molecular simulation approaches on M.HhaI-DNA-S-adenosylhomocysteine ternary complex. The results demonstrate that the nonspecific binding of DNA to M.HhaI is initially induced by electrostatic interactions. Differences in chemical environment between the major and minor grooves determine the orientation of DNA. Gln237 at the target recognition loop recognizes the GCGC base pair from the major groove side by hydrogen bonds. In addition, catalytic loop motion is a key factor during this process. Our study indicates that base flipping is likely to be an “induced-fit” process. This study provides a solid foundation for future studies on the discovery and development of mechanism-based DNA methyltransferases regulators.

## 1. Introduction

DNA methylation at the position 5 of cytosine, which is closely related to development and differentiation, genome stability, genomic imprinting, X-chromosome inactivation, and silencing of retrotransposons [1–4], is commonly found in bacteria, plants, and mammalians. Hypermethylation of specific genes is found to be closely related to many malignant diseases [5]. DNA methylation is catalyzed by DNA methyltransferases (DNMTs), which have been identified in at least 16 kinds of bacterial DNMTs [6] and 3 kinds of mammalian ones. Crystal structures of different DNMTs [7, 8] show that the catalytic domains of these methyltransferases are relatively conserved. Recent studies demonstrate that these enzymes also share a similar catalytic mechanism.

**Figure 1:**
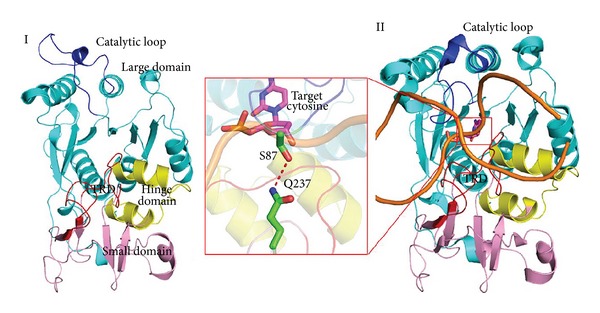
Two conformations of M.HhaI. (I) The structure of the M.HhaI-SAM binary complex (PDBID:2HMY) shows the inactive status of M.HhaI. The large, hinge, and small domains are colored marine cyan, yellow, and pink. (II) Structure of the M.HhaI-SAH-DNA ternary complex (PDBID:2HR1) and the active status of this enzyme. Carbon atoms of target cytosine are colored magenta. The target recognition domain (TRD) and catalytic loop in both structures are colored red and blue, respectively. Flipped cytosine, Gln237, and Ser87 are shown using sticks.

HhaI methyltransferase (M.HhaI) belongs to restriction-modification systems of bacterial DNMTs [9] and methylates certain CpG sequences specifically. To access the target base and modify the genetic information, M.HhaI flips the target base out of the DNA double helix during the catalytic process. Base flipping was first discovered by Cheng et al. in the cocrystal structure of cytosine-5 DNA methyltransferase binding to DNA [8]. Structures of M.HhaI can be divided into three parts: a large domain (residues 1–193 and 304–327), a small domain (residues 194–275), and a hinge region (residues 276–303) [10–12] (Figure 1). The target recognition domain (TRD) is located in the small domain and plays an important role in recognizing cognate GCGC base pairs. The catalytic loop (residues 81–100), a very flexible motif in the large domain, is located opposite the TRD. Based on the conformations of the catalytic loop, these structures can be classified into two distinct classes: the catalytic inactive (Figure 1(I)) and catalytic active (Figure 1(II)) forms. Various assays, including fluorescence [13], NMR [14], and molecular dynamics simulation, have been used to investigate M.HhaI, and valuable information on different aspects of the base flipping process has been obtained. (1) M.HhaI binds to the nonspecific DNA or cognate DNA (DNA containing GCGC nucleotides) with different affinities [15, 16], and the DNA sequence plays an important role in substrate recognition and conformational transition of the catalytic loop. (2) Base flipping is involved in extensive protein conformational changes, including closure of the catalytic loop (residues 81–100 of M.HhaI) [17], target base flipping, and correct assembly of the active site [18]. (3) The target base preferably rotates out of the double helix through a major groove path [19] because of interactions between the HhaI methyltransferase and the backbone of the cognate DNA [20]. However, these studies mainly focused on flipped base and surrounding residues in active state; these states may not adequately describe nonspecific binding pattern and following sequence recognition process. Thus, understanding the detailed process of structural rearrangement of catalytic loop and the relationships between target sequence recognition and catalytic loop reorganization remains challenging.

To gain new insights into the conformational transition of M.HhaI, we performed a mechanistic investigation on the dynamic transition of this enzyme using a combination of molecular docking, conventional molecular dynamics (MD) simulation, and metadynamics simulation. The DNA-M.HhaI- (open form-) S-adenosylhomocysteine (SAH) ternary complex built by protein-DNA docking model is used as the starting structure and then optimized by conventional MD simulation. Subsequently, metadynamics simulation is employed to monitor the motion of catalytic loop. The results show that DNA binds to a shallow pocket close to the catalytic loop before it falls into a cleft between the TRD and the catalytic loop. DNA binds to this nonspecific binding site and evokes the conformational change of residues at the tip of this motif. Target recognition loops I and II detect the target DNA and facilitate target base flipping by destabilizing hydrogen bonds between base pairs. Our study shows the nonspecific binding patterns of DNA, sequence recognition process of M.HhaI, and conformational reorganization of the catalytic loop. We propose that DNA evokes the conformational change of M.HhaI, which then selects the target cytosine to fit into its catalytic pocket actively. Understanding the mechanism of DNA recognition process in base flipping at the atomic level is of great help to future researchers. This study explains DNA recognition process in atomic detail and will aid the future discovery and development of mechanism-based DNMT regulators.

## 2. Materials and Methods

### 2.1. Starting Structure for Simulation

Crystal structures of the binary complex (PDB ID: 2HMY [7]) were used to construct the protein model, in which all water molecules were removed. For ligands in the crystal, SAM was converted into SAH by simply removing the methyl group attached to the sulfur atom, whereas solvents and other molecules were deleted. DNA used in this simulation was generated by the 3D-DART server [21], and the sequence employed was identical to that in the M.HhaI-DNA-SAH complex (PDBID:2HR1). To place a piece of cognate DNA in its “nonspecific” site, protein-DNA docking was employed. Docking was performed on the PatchDock web server [22]: the prepared protein was used as the receptor and the B-form DNA generated by 3D-DART was used as the ligand.

Little is known about nonspecific binding sites and the binding poses of M.HhaI. To find an appropriate starting point, biomolecular docking was employed. This method is commonly used to gain structural insights into macromolecule structures that X-ray crystallography or NMR spectroscopy cannot elucidate [23]. Patchdock [24, 25] is a geometry-based molecular docking algorithm that can be used for protein-protein, protein-ligand, or protein-DNA docking. We docked B-form DNA into proteins through the Patchdock web server. Fifty different poses were downloaded from the server. We then separated these poses into two categories: (1) DNA approaching the TRD and (2) DNA approaching the catalytic loop. Combining the structures of M.HhaI at different states and the NMR experiment results (see Supporting Information, available online at http://dx.doi.org/10.1155/2014/304563), we chose the top scored poses in category two as our initial structures.

### 2.2. Conventional MD Simulation

This initial structural model was prepared using Charmm27 all-atom force field by pdfgen. Then, the ternary complex was embedded into an explicit TIP3P water molecule box with 10 Å widths. 61 Na^+^ and 39 Cl^−^ ions were added to this box to ensure charge neutrality. Finally, the concentration of NaCl was adjusted to 0.11 mM by the Autoionization plug-in (version 1.3).

The system described above of ~62,000 total atoms underwent 5,000 steps of water molecule minimization keeping all heavy atoms of protein, DNA, and SAH fixed, 2,000 steps of minimization with only the protein backbone fixed to allow protein side chains to relax, and another 5,000 steps of minimization without any constraint on the system. The energy-minimized system was gradually heated to 300 K in 50,000 steps at a rate of 5 K per 1,000 steps at constant volume using a Berendsen thermostat [26]. The L-J potential cutoff of molecular dynamics simulation was set to 14 Å. Then, the whole system was equilibrated with unbiased MD simulations for 5 ns under NPT conditions.

### 2.3. Targeted MD Simulation (TMD)

Targeted molecular dynamics (TMD) simulation was employed to guide a set of atoms moving from its initial to a given target structure by means of the steering forces. In this experiment, the transitions of M.HhaI from inactive to activate state were driven by applying RMSD restraints with a force constant of about 1 kcal/mol/Å^2^ to each heavy atom of the catalytic loop (residues Cys81–Leu100). The offset parameter of RMSD decreased by about 0.027 Å per ps until it reached zero deviation. The total TMD simulation lasted for 2 ns.

### 2.4. Path CV Based Well-Tempered Metadynamics

Metadynamics [27] in its new well-tempered variant [28] was used for free energy calculation. The free energy at time (*t*) was defined using the following formula:

(1) $$\begin{array}{c} F\left(s,t\right)=-\frac{T+\Delta T}{\Delta T}V\left(s,t\right), \end{array}$$

where *F*(*s*, *t*) stands for the free energy at time *t*, *V*(*s*, *t*) is the bias potential added to the system, and *T* is the temperature used for this simulation. Δ*T* is the difference between the fictitious temperature of the CV and the temperature of the simulation. The bias potential is made up by the sum of the Gaussians deposited along the trajectories of the CVs.

To trace the path, two variables *s*(*R*) and *z*(*R*) were introduced as [29]

**Scheme 1:**
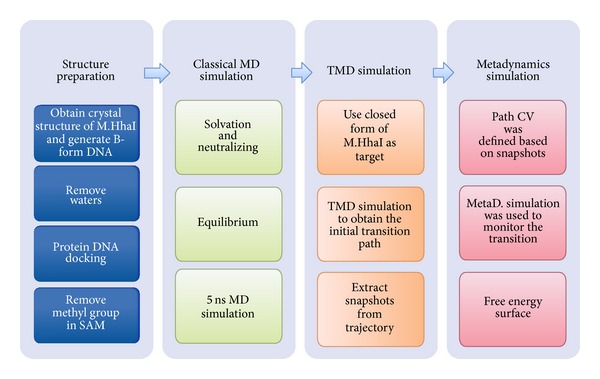
Molecular simulation protocol.

(2) s(r)=∑l=1Ple−λ||S(r)−S(l)||2∑l=1Pe−λ||S(r)−S(l)||2,z(r)=−1λln⁡⁡(∑l=1Pe−λ||S(r)−S(l)||2),

where the distance between the current position *S*(*R*) and the reference frames of the path *S*(*l*) is calculated using a DMSD metric after alignment to the reference using a rototranslation matrix [30]. To define *s*(*R*), 31 reference frames were selected from the TMD trajectory. Heavy atoms of the catalytic loop (residues Cys81–Leu100) were selected for DMSD calculation. In addition, nonhydrogen atoms of an alpha helix immediately after the catalytic loop (residues 102–120) were used for alignment. The mean interframe RMSD of these frames is 0.46 Å^2^. According to the relationship between mean RMSD and *λ*, the *λ* value was set to five. We performed metadynamics only in the space of *s*(*R*) whereas *z*(*R*) was constrained to 3 Å^2^. The hill height was set to 1.5, and the bias factor was set to 8. NAMD 2.8 [31] with the Plumed 1.3 plug-in [32] was employed for all simulations. A summary of simulation protocol is surmised in Scheme 1. Detailed parameters, preliminary simulations, and postprocessing protocols are listed in Supporting Information.

## 3. Results and Discussion

### 3.1. Biased MD Simulations

In order to avoid clash between modeled structure and surrounding solvents, a short conventional MD simulation was performed. According to the RMSD profile relevant to the starting structure along MD trajectory, the complex structure appeared to have reached a stable state after 4 ns equilibration, where the RMSD value converged to a value around 4.0 Å (shown in Supplementary Information). Given the limitation of standard molecular dynamics (MD), enhanced sampling was employed to overcome the energy barrier. Among the techniques currently available, metadynamics has shown to be useful in studies of conformational changes of proteins [33], peptide folding [34], or chemical reactions [35]. In this study, we performed metadynamics in its new variant, named well-tempered metadynamics, which allows reconstruction of the free-energy profile of the process of interest by adding an adaptive bias on a selected number of collective variables (CVs) [28]. Thus, choosing an appropriate CV is vital to successful metadynamics simulations.

Considering both protein and DNA participated in DNA recognition process, we employed two CVs to describe the transition path of catalytic loop and cognate GCGC sequence, respectively. Path CV is very useful tools which transform the high-dimensional phase space to a one-dimensional description [36]. As a result, we employ RMSD of heavy atoms to demonstrate the motion of catalytic loop. On the other hand, we choose the distance between center of mass (COM) of GCGC and COM of TRD as another CV defines the transition path. Under the acceleration of metadynamics, this loop is able to move from one free-energy minimum state to another, thereby overcoming the large free-energy barriers that are encountered during the transition process.

### 3.2. DNA Migrates into a Binding Cleft between TRD and Catalytic Loop as a Result of Electrostatic Attraction

**Figure 2:**
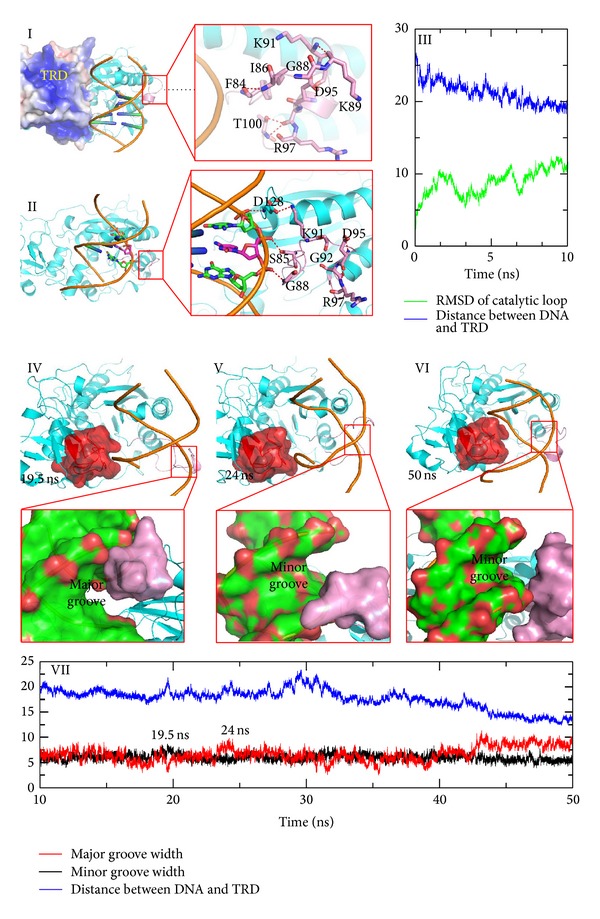
DNA migrates into a cleft between TRD and catalytic loop. Nonspecific DNA binding initiating the conformational transition of the catalytic loop is shown in the top. (I) Structure of the initial model. The open state of the catalytic loop is stabilized by hydrogen bonds between the main chain atoms. The electrostatic surface is generated by APBS and the positively charged area is colored blue. (II) Snapshots extracted from the MD simulation. Residues at the tip of the catalytic loop move toward the DNA backbone because hydrogen bonds between the side chain atoms and the DNA backbone replace the original hydrogen bond network. (III) RMSD and distance plot along trajectory. The distance COM of the target recognition loop (residues 230–260) and COM of GCGC are plotted in blue lines, whereas the RMSD values of the catalytic loop are plotted in light green lines. A series of snapshots that describe DNA rotation in a cleft between the TRD and the catalytic loop is shown at the bottom. (IV)–(VI) Different snapshots extracted from the trajectory. TRDs are represented with a red extended surface; major and minor grooves are also highlighted using the extended surface. (VII) Groove width and distance plot determined from the MD simulation. The major width plot is colored red, the minor groove is colored black, and the distance is colored blue.

During the simulation, DNA initially enters into the binding cleft between the TRD and the catalytic loop. This process can be divided into two phases. In the first phase (0 ns to 10 ns), DNA induces a conformational change of M.HhaI, leading to formation of a binding cleft between the TRD and the catalytic loop. As shown in Figure 2(III), the distance between the cognate GCGC base pair and the two target recognition loops (residues 233–240 and 252–258) [37] decreases by about 7 Å over 10 ns. When the DNA moves towards the TRD, the direction of the catalytic loop changes simultaneously as shown in Figure 2(II). Residues at the tip of catalytic loop, such as Ser85, Ser87, and Lys89, move towards the TRD and evoke rearrangement of the entire catalytic loop. The RMSD profile confirms that the catalytic loop undergoes a distinct conformational change (Figure 2(III)). When the DNA and catalytic loop move towards the TRD, a cleft between the TRD and the catalytic loop formed. DNA enters this cleft and interacts with TRD through the phosphodiester backbone. Cleft formation and DNA binding may be largely attributed to electrostatic attraction, because the TRD of this enzyme is a positively charged motif (as shown in Figure 2(I)), whereas the phosphor group at the DNA scaffold is negatively charged.

In the second phase (10–50 ns), DNA is accommodated into the binding cleft by adjusting its groove width and orientation. As shown in Figure 2(VII), both the major and minor groove widths of DNA fluctuate as the simulation proceeds. The groove width profile and snapshots derived from the trajectory demonstrate that the groove width affects the location of DNA and the catalytic loop conformation. The average minor groove width increased as the major groove narrowed at 19 ns (average groove width of the GCGC motif is 3.2 Å) and the catalytic loop approached the major groove of the GCGC sequence. The distance between DNA and the TRD increased to accommodate the TRD (Figure 2(IV)). At about 24 ns, the minor groove narrowed but the volume of the major groove increased (shown in Figure 2(V), average groove width, 9.8 Å). As the groove width changed, the catalytic loop gradually penetrated into the minor groove of DNA (RMSD value increased) and the major groove was accommodated into the TRD of M.HhaI. During this period, DNA is rotated about 45° along with the groove width fluctuation. At the end of this period (about 50 ns), DNA adopts a relatively stable orientation with the major groove facing the TRD and the minor groove facing the catalytic loop (Figure 2(VI)).

### 3.3. The Target Recognition Domain Recognizes Cognate DNA by Hydrogen Bonded to the GCGC Base Pair in Both the Target and Complementary Strands

**Figure 3:**
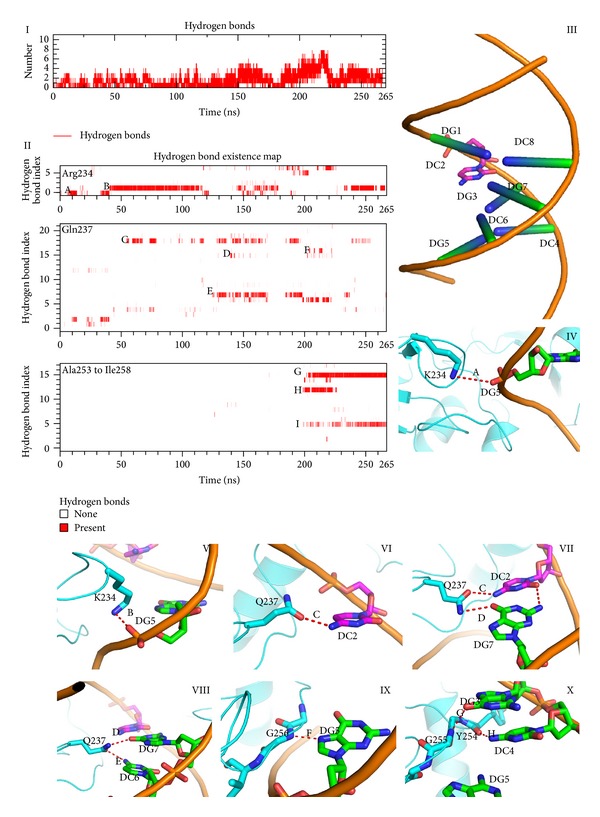
Hydrogen bonds between GCGC motif and target recognition loop. (I) Hydrogen bond number plot along the trajectory. (II) Hydrogen bond existence map of Arg234, Gln237, and target recognition loop (II). (III) Illustration of the notions used in Figures 4, 5, and 6. (IV)–(X) Highlighted hydrogen bonds in the existence map.

Binding and recognition of the target GCGC site in DNA is a key event that occurs before base flipping [38]. Formation of hydrogen bonds may play an important role in this recognition process. Here, we monitored the hydrogen bonds number and existence map along the trajectory. As shown in Figure 3(I), the hydrogen bonds number between TRD and GCGC increased along the trajectory. The existence map, which presents the hydrogen bond formation process, indicates that Gln237 detects the target cytosine (DC2) and GC bases in the complementary strand, whereas target recognition loop II identifies DG3 and DG4 in the target strand and DG5 in the complementary strand (Figure 3(III–X)). Similar results were found in the NMR and fluorescence experiments [15, 39]. The sequence specificity of C5-MTases is largely attributed to two recognition loops located in the target recognition domain [39]. Furthermore, target recognition loops I and II recognize different parts of cognate GCGC sequences. Target recognition loop II recognizes DG3 and DG4, which are located at the 3′ end of the target cytosine, by hydrogen bonding to guanine and cytosine bases [15, 39]. By contrast, target recognition loop I recognizes the 5′ end of the target site. Gln237 plays an important role in this process; thus Q237A mutations show significantly decreased base flipping rates [40, 41].

### 3.4. Target Recognition Loops and the Catalytic Loop Facilitate Base Flipping by Evoking and Stabilizing the Preflipping Statue

**Figure 4:**
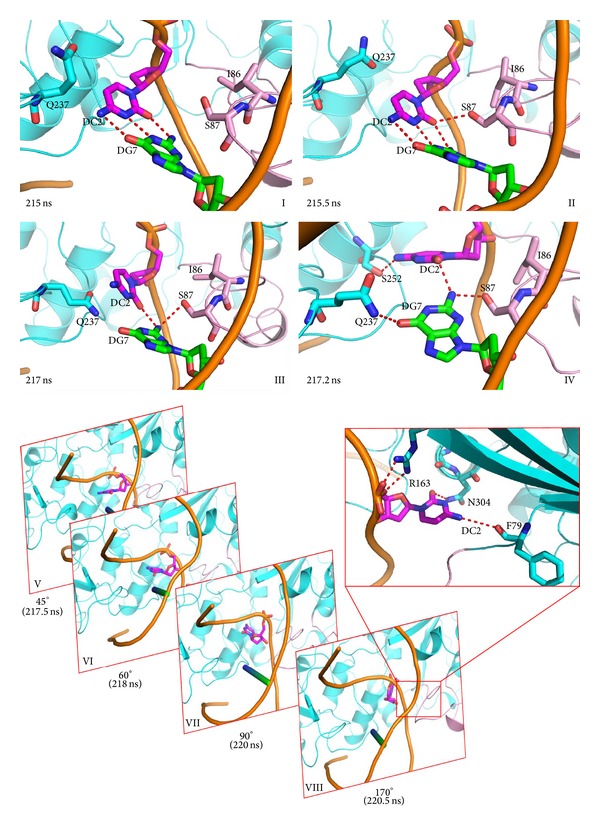
Base preflipping and base flipping process. (I)–(IV) Preflipping states evoked by the pushing of the DNA backbone by the catalytic loop using Ile86 and Ser87. (V)–(VII) The target cytosine flips out of the DNA double helix from the major groove side. Time and flip angles are noted at the bottom of the snapshots.

After the GCGC sequence is recognized by M.HhaI, the distance between DNA and the TRD achieves its minimum at about 10 Å. Residues at the tip of the catalytic loop sense the translocation of the DNA backbone and rearrange its conformation to move along with the DNA scaffold. Ile86 and Ser87 are inserted into the minor groove of DNA because of reorganization of the catalytic loop. DNA backbone twisting results in increased distances between the target cytosine and the complementary guanine. The hydrogen bonds between the G:C pair are impaired, and the original hydrogen bonds loss force guanine or cytosine hydrogen bond to surrounding Ser87, Gln237, and Ser252 (Figure 4(I–IV)). After the target base is stabilized by surrounding residues, cytosine rotates about 15° out of the DNA double helix. This observation is coincident with previous molecular dynamics simulations [42], fluorescence tracking [43], and NMR experiments [44].

Preflipping leads to the loss of hydrogen bonds *π*-*π* staking; as a result, this state is not very stable and the cytosine was quickly flipped out of the double helix from the major groove (Figure 4(V–VIII)). After base flipping, residues surrounding the flipped cytosine, such as Phe79 and Gln304, stabilized the flipped status by bonding hydrogen to the cytosine base ring. This “major groove pathway” is also observed in the crystal structures, molecular dynamic simulations [19, 45], and NMR experiments [46]. On the other hand, base flipping is observed before catalytic loop is fully close, and the dynamic properties are a very important factor that affect base flipping process. This observation is coincident with results of NMR, molecular dynamic studies [16, 20, 47, 48], and mutation experiments [49] and previous research [9, 50, 51].

### 3.5. Conformations Transition of Catalytic Loop

**Figure 5:**
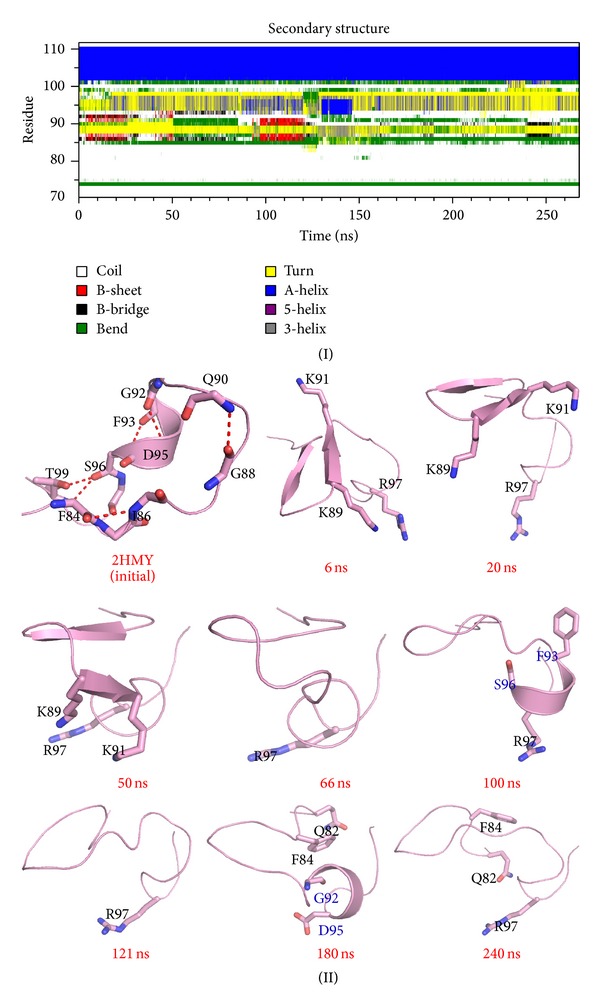
Conformational transition of catalytic loop during simulation. (I) Secondary structure profile of the catalytic loop. (II) Snapshots extracted from the MD simulation. In the 100 and 180 ns snapshots, the beginning and end of the short helix are colored blue. Carbon atoms are colored pink, and other atoms are colored using default settings in PyMol.

The catalytic loop is a very flexible motif and has an important function in base flipping, catalytic pocket formation, and methyltransfer reactions [48]. Molecular dynamics simulations [45], crystallography studies [19, 52, 53], and mutation experiments [51, 54, 55] show that the dynamics of conformational rearrangement occurring in the catalytic loop are closely related to the base flipping process. We monitored the transformation of the secondary structure between Pro70 and Leu110 (Figure 5(I)) to observe conformational changes in the catalytic loop. M.HhaI adopts an open conformation in the solution (PDBID:2HMY) [15]: the catalytic loop stays away from the TRD and the heteroatom of the polar side chain in the catalytic loop points opposite to target recognition loops. This conformation is stabilized by the hydrogen bonds between the main chain atoms. When M.HhaI binds DNA in a nonspecific manner, residues at the tip of the catalytic loop, such as Lys89, Lys91, and Gln90, flip their side chain and approach the DNA backbone gradually (about 6–50 ns) (Figures 2(II) and 2(VI)), and the number of hydrogen bonds between the main chains decreases. Then, catalytic loop undergoes an extensive conformational change: (1) unfolding of short helix from Lys91 to Asp95 to a coiled structure (snapshot at 6 ns in Figure 5(II)), (2) gradual formation of antiparallel beta-pleated sheet between Ser85 and Gln90 (snapshot at 20 ns in (Figure 5(II))), (3) rotating of the *β*-sheet of Ser87 to Asp95 around the *β*-sheet axis by about 90° (snapshots at 50 and 66 ns in Figure 5(II)), (4) refolding of a helix between Gly92 and Ser96 (snapshot at 180 ns in Figure 5(II)), and (5) main chain atoms between Gln82 to Ser85 changing their orientation (snapshot at 240 ns in Figure 5(II)). However, Gly98 preserves its conformation, and the phi and psi angles between Gly98 and its adjacent Thr99 remain unchanged. As Matje et al. mentioned, this “hinge” may aid the refolding process because the mutation of Gly98 is believed to affect the base flipping process [51]. Besides, the orientation of guanidyl of Arg97 changes along with the unfolding of short helix from Gly92 to Ser96 and refolding process of the short helix. Helix unfolding forced the guanidyl of Arg97 to leave the DNA backbone (snapshot at 100 ns in Figure 5(II)). Nevertheless, electrostatic attraction induced Arg97 to move toward a phosphor group of the DNA (snapshot at 121 ns in Figure 5(II) and following refolding of the Phe93 to Ser96 segments.

**Figure 6:**
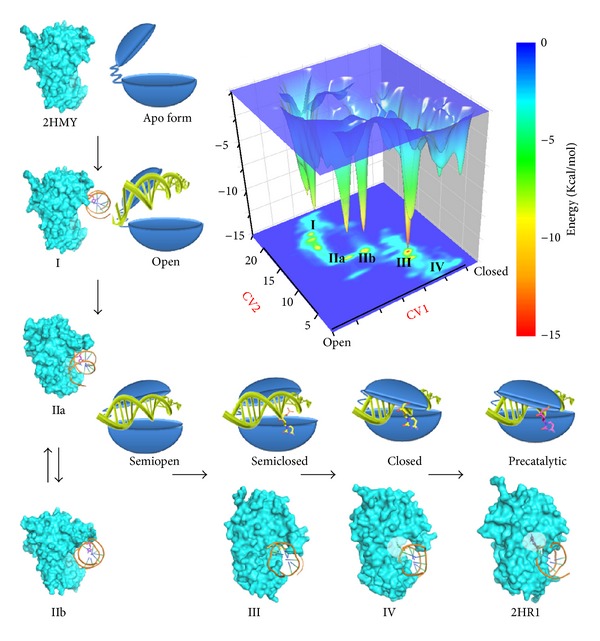
Inactive to active form transition. Free energy profile acquired from metadynamics simulation is shown in upper right corner. To describe the open to closed conformational transition of catalytic loop, path CV (CV1) [29] is used. In addition, we used a distance CV (CV2) that measures the distance between the COM of the GCGC base pair and the COM of the two target recognition loops. No bias is added to the distance CV, and the statistics collected during the metadynamics simulations are used to generate the final FES using the reweighting protocol [60] according to Limongelli's approach [61]. Proposed transition path from inactive form (2HMY) to active form (2HR1) is represented in an “L” shaped manner. (I)–(IV) show different basins obtained from the metadynamics simulation. Basins IIa and IIb are noted because their protein conformations are similar. Proteins are represented by an extended surface. In snapshots (IV) and (2HR1), residues around the flipped base are semitransparent to aid visualization of the flipped base. Schematic diagrams of these different states are placed beside the corresponding snapshots. Proteins are represented in a Pac-Man-like form [62] with the upper cap and lower base connected by a hinge. DNA is represented using the double helix.

Combining the conformational reorganization of the catalytic loop, five basins were acquired from the free energy surface and the snapshots extracted from the MD trajectory. We propose that enzymes undergo “open,” “semiopen,” “semiclosed,” and “closed” states to accomplish the entire transition and facilitate the base flipping process (Figure 6), and the enzyme uses these different conformations to sense DNA binding and screen DNA sequence, find cognate GCGC, and flip the base, respectively.

### 3.6. The Mechanism of M.HhaI Screens Different DNA Sequence

When M.HhaI binds to the DNA loosely in the “semiopen” state, the DNA twists and translocates. This binding pattern provides a platform through which the enzyme can search for its target sequence [15]. Conformations (IIa) and (IIb) share similar probabilities, as shown in the free energy surface (Figures 6(IIa) and 6(IIb)); basin IIb is approximately 0.5 kcal/mol deeper than basin IIa. Thus, both the major and minor grooves have the opportunity to face the TRD or the catalytic loop of M.HhaI. The DNA rotates around the screw axis of the double helix at approximately 45° when the complex transforms from a IIa-like pattern to a IIb-like one. If a cognate sequence is detected by the target recognition loops, the GCGC will create a hydrogen bond with these loops and decrease the dynamics of the DNA backbone. Finally, DNA rotation is hindered and the recognition process begins. However, if the bases located in the major groove cannot be recognized by the TRD, the major groove leaves the TRD through “rotation-coupled sliding along the DNA helix,” which is a general phenomenon found in DNA glycosylases and other similar enzymes [56]. Therefore, we speculate that catalytic loop is responsible for evoking DNA rotation and searching for the appropriate DNA sequence simultaneously when a segment of a double stranded DNA molecule or a plasmid binds to M.HhaI because the energy barrier between (IIa) and (IIb) is about 7.2 Kcal/Mol.

Recognition of the cognate sequence is an important process prior to base flipping and methyltransfer [15]. After the target sequence is detected by M.HhaI, the catalytic loop moves closer to a TRD, and the system enters basin (III) (Figure 6(III)), which is deeper than any other minima. Experimental data indicate that the side chains of Gln237 and Arg240 are vital to evoke DNA methylation and base flipping; however, according to the metadynamics simulation, their role in the recognition phase may be different. Experiments suggest that whether or not guanine is replaced by other purine base or purine-like substrates, the base flipping rate is similar as long as hydrogen bonds between Arg240 and the base are preserved [57]. While both Q237 mutating and GCGC sequence missing abolished the catalytic activity of M.HhaI, in our simulation, the hydrogen bonds of Gln237 and GCGC are distributed within 150–200 ns (Figure 3). These hydrogen bonds include the target cytosine, the orphan guanine in the complementary strand, and cytosine 5′ between the side chains of Gln237. During this period, the distance of GCGC and the target recognition loops decreases. The driving force of GCGC motif approaching TRD is speculated to be polar interactions. Thus, besides stabilizing the flipped cytosine, Gln237 may function as a probe to detect CG binucleosides in the target and complementary strands.

### 3.7. Base Flipping: An Induced-Fit Process

Different hypotheses have attempted to demonstrate the base flipping process. Research shows that closure of the catalytic loop occurs after base flipping [9, 58] and that the enzyme utilizes the hydrogen bonds between Gln237 and Ser87 to lock the flipped base. Other research studies indicate that target base flipping and closure of the mobile catalytic loop occur simultaneously [17]. Our results are in agreement with the second theory, which is also known as the “induced-fit” hypothesis. This model was first presented in 2004, in which tight DNA binding is thought to be coupled with base flipping and protein loop rearrangement [11]. Subsequent fluorescence experiments [18] and molecular dynamic simulations [59] also support this theory, which demonstrates that loop rearrangements are directly coupled with base flipping. Our simulations show that the induced-fit process of DNA-protein recognition begins immediately after DNA binding to a nonspecific binding site. The negatively charged DNA backbone triggers conformational rearrangement of the catalytic loop. Then, a DNA binding cleft emerges after the catalytic loop changes its conformation. Formation of this cleft provides the enzyme with the ability to bind to DNA loosely and search for its target sequence and target base. Selected base or sequences make contact with the TRD, whereas other sequences are rejected by DNA movement. As target bases fit into the TRD, the phosphate backbone of the DNA initiates another conformational rearrangement of the catalytic loop. While the target is flipped out of the DNA double helix, the DNA approaches the TRD even as the catalytic loop is not fully closed. Subsequently, another extensive conformational transition of the catalytic loop elicited by base flipping contributes to the folding of the catalytic pocket and stabilization of the flipped cytosine.

## 4. Conclusion

Base flipping appears in a number of systems and enzymes, and debates regarding the detailed process and mechanism of this phenomenon persist. Here, we performed metadynamics simulation on the M.HhaI-SAH-DNA ternary complex to provide a better understanding of this interesting phenomenon. Consistent with previous experimental findings, we found that both protein and DNA play important roles in nonspecific binding, DNA sequence recognition, and the flipping process. Moreover, during the open to closed transition process, we captured a series of intermediates, the transition process into four phases according to the free energy landscape constructed based on MD simulation, and the transition process can be divided into four phases. In each phase, key residues found in the simulation coincided with data from previous experiments. Combining these findings, we proposed an “induced-fit” model to illustrate the base flipping process in M.HhaI. The results of our simulations demonstrate base flipping at the atomic level and help elucidate the mechanism underlying the base flipping process.

## Supplementary Material

Preliminary metadynamics simulation, different snapshots of flipped bases, and methods used for trajectory processing are listed in supporting information.
