# Supplementary material for: Metadynamics Simulation Study on the Conformational Transformation of HhaI Methyltransferase: An Induced-Fit Base-Flipping Hypothesis
Source: Biomed Res Int. 2014 Jun 19;2014:304563. doi: 10.1155/2014/304563 (PMC4090504; doi:10.1155/2014/304563)
Supplement: Supplementary file 1 — Preliminary metadynamics simulation, different snapshots of flipped bases, and methods used for trajectory processing are listed in supporting information. [file 304563.f1.pdf]

# Supporting Information

## 1. Constructing the starting structure

Comparing crystal structure of M.HhaI with and without DNA1, 2 (PDBID:1hmy and PDBID:2hmy), we find that: the RMSD of overall structure and TRD domain was less than 0.4Å, while the RMSD of Catalytic loop was 1.07Å.(shown in Figure S1) Furthermore, NMR experiments also showed that, non-specific DNA evoked chemical shift of residues in catalytic loop instead of TRD. Additionally, because catalytic domain in DNMT1 and M.HhaI share a similar three dimensional structure3, we check the crystal structure of DNMT1-non-specific DNA complex. In the crystal structure, catalytic loop hydrogen bonded to phosphor group of non-specific DNA by M1235, N1236, R1237 and R1241 (not shown). As a result, we choose the top-scored pose of the second categories as our initial model. In our model, amino acids with polar side chains, such as Ser85, Ser87 and Lys89, touch DNA phosphate backbone. This resembles the binding pattern when DNMT1 binding to non-specific DNA.

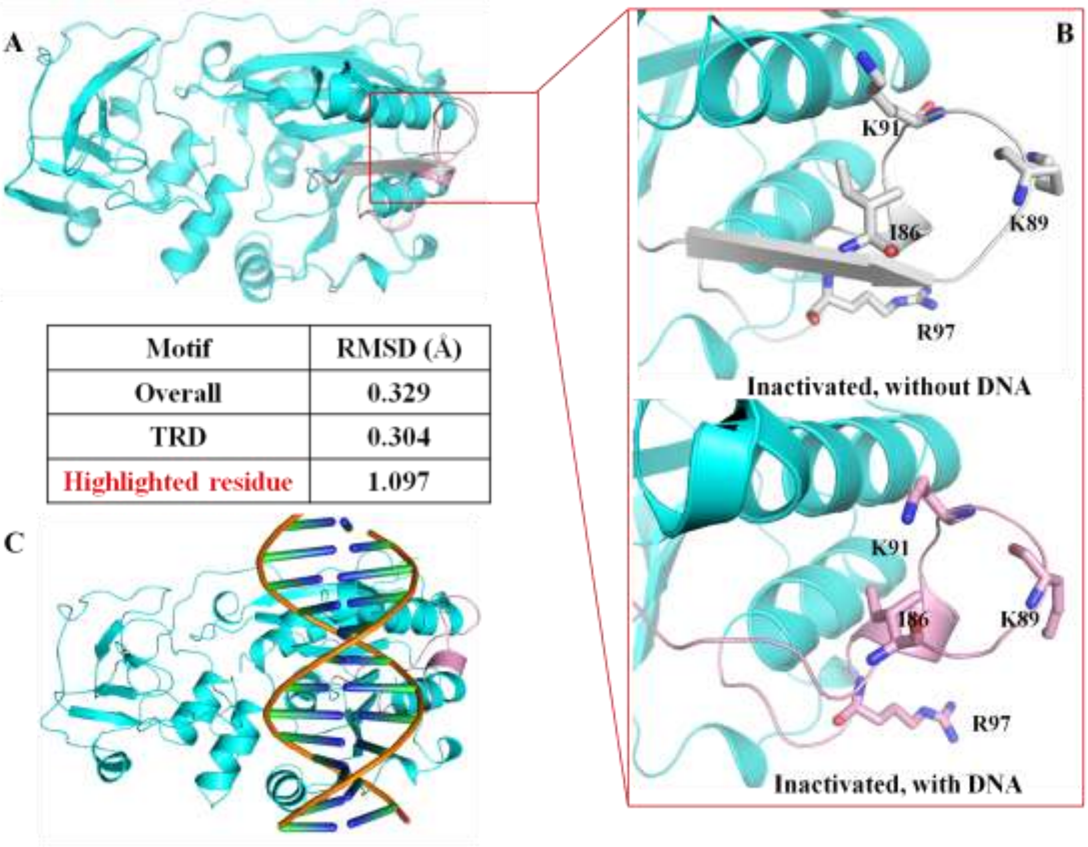


| Motif | RMSD (Å) |
|---|---|
| Overall | 0.329 |
| TRD | 0.304 |
| Highlighted residue | 1.097 |

**Figure S1. The starting structure. A.** Comparing M.HhaI-SAM binary complex with and without DNA. **B.** Detailed conformational differences of key residues in catalytic loop. Catalytic loop of M.HhaI binary structure without DNA is colored white, while catalytic loop in binary structure with DNA is colored pink. Other part of M.HhaI is colored cyan. RMSD comparisons of different motifs are listed in table. **C.** Three dimension structure of starting structure.

## 2. Post processing protocol

Trajectory analysis is performed using different tools. We use CatDCD converted original DCD type trajectory into TRR and PDB format. RMSD of catalytic loop and Distance map was plotted by g_rms and g_dist tool in Gromacs 4.5.5[4]. Hydrogen bond existence map and Hydrogen bond number map was plotted using g_hbond tools in Gromacs. We employ Curves+[5] to monitor the groove width. Elctrostatic surface is generated by using APBS[6] plug-ins in PyMol. The secondary structure profile of M.HhaI was generated using do_dssp in Gromacs 4.6. Then, we use xpm2ps converted xpm format into PostScript file.

Three dimensional structures were made by PyMol. Free energy profile was generated using the RGL module in the R program[7]. The other plots are drawn using the XmGrace (http://plasma-gate.weizmann.ac.il/Grace/) plotting tool. The VMD program[8] was used for trajectory visualization.

## 3. Preliminary Metadynamics Simulations

Choosing proper collective variables (CVs) is vital to metadynamics simulation. Because catalytic loop motion was a very complex process, we tried six different CVs and their combination:

(1) The distance between the center of mass (COM) of the catalytic loop and two target recognition loops;

(2) The distance between amino group in the side chain of Gln237 and carboxyl group of Ile86;

(3) Different psi and phi angle combinations of residues from 81 to 89, in order to monitor the loop formation process;

(4) Alpha helix formation between residue 82 and 84;

(5) Contact map[9] between GCGC motif and Target recognition loop of M.HhaI.

We also use TMD trajectory to test these CVs and their combinations (in Table S1). Plotting the value of these CVs along TMD trajectory, we find they can separate initiation and ending successfully. However, no obvious conformational change of catalytic loop can be found in following metadynamic simulation. All these runs are considered unsuccessful. Since the complexity of the simulation system, it requires a CV to describe the motion of catalytic loop. Thus, we tried DMSD as CV to define the transition path. We also tried different ways to describe this CV: using atoms in the backbone of catalytic loop or heavy atoms in the catalytic loop. Finally, the result shows that using all non-hydrogen atoms could successfully drive the catalytic loop moving from open to closed form only.

**Table S1. Details of CV definition**

| Run | CV Type | Atoms | Groups |
|---|---|---|---|
| | Distance | COM of backbone atoms in catalytic loop (residue 81-100) | 1 |
| **1** | Distance | COM of target recognition loop (I and II) | 2 |
| | Torsion | Target base flipped angle* | - |
| | Distance | amino group in the side chain of Gln237 | 1 |
| **2** | Distance | carboxyl group of Ile86 | 2 |
| | Torsion | Target base flipped angle | - |
| | Torsion | Phi angle of residue 84 | 1 |
| **3** | Torsion | Phi angle of residue 82 | 2 |
| | Torsion | Target base flipped angle | - |
| | Torsion | Phi angle of residue 88 | 1 |
| **4** | Torsion | Phi angle of residue 89 | 2 |
| | Torsion | Target base flipped angle | - |
| | Alpha | Residue from 82 to 84 | 1 |
| **5** | Alpha | Residue from 91 to 96 | 2 |
| | Torsion | Target base flipped angle | - |
| | CMAP | Backbone atoms in target recognition loop I (residue 233-240) and nitrogen or oxygen atom of base in GCGC | 1 |
| **6** | Distance | COM of Backbone atoms in GCGC motif and COM of residue 87 to 91 | 2 |
| | Torsion | Target base flipped angle | - |

*Target base flipped angle: this angle was defined according to reference[10].

# 4. Flipped base in semi-closed form

Base flipping take place at about 230ns, and this progress is rapid. After target base flipped, hydrogen bonds between Arg163, Arg165, Phe79 and target cytosine is observed. Overlapping the snapshot of 265ns with the ternary complex (PDBID: 2HR1), the RMSD value of the catalytic loop is 2.703 Å, and the RMSD of overall structure is 1.521 Å (shown in Figure S2). As a result, our starting model and following simulation is reasonable. Whiel there are also some deficiency in it: since the ending of this

simulation, we have not observed the hydrogen bonds between Arg240 and Guanine 3' to target cytosine, and the formation of catalytic pocket. On the other hand, target cytosine is not stable (shown in Figure S3). Target cytosine rotate around an axis which connecting N1 and N6 90 degrees, then this cytosine tilts about 60 degrees, and the N6 atom hydrogen bonded to phosphor atom of nucleoside 5' to cytosine. Additionally, Amide group of Gln237 have not hydrogen bonded to Ser85. As a result, the catalytic loop has not proceeded to closed form, and the catalytic pocket of M.HhaI has not formed. We consider these insufficiency is caused by limited time scale and force field parameter accuracy[11, 12].

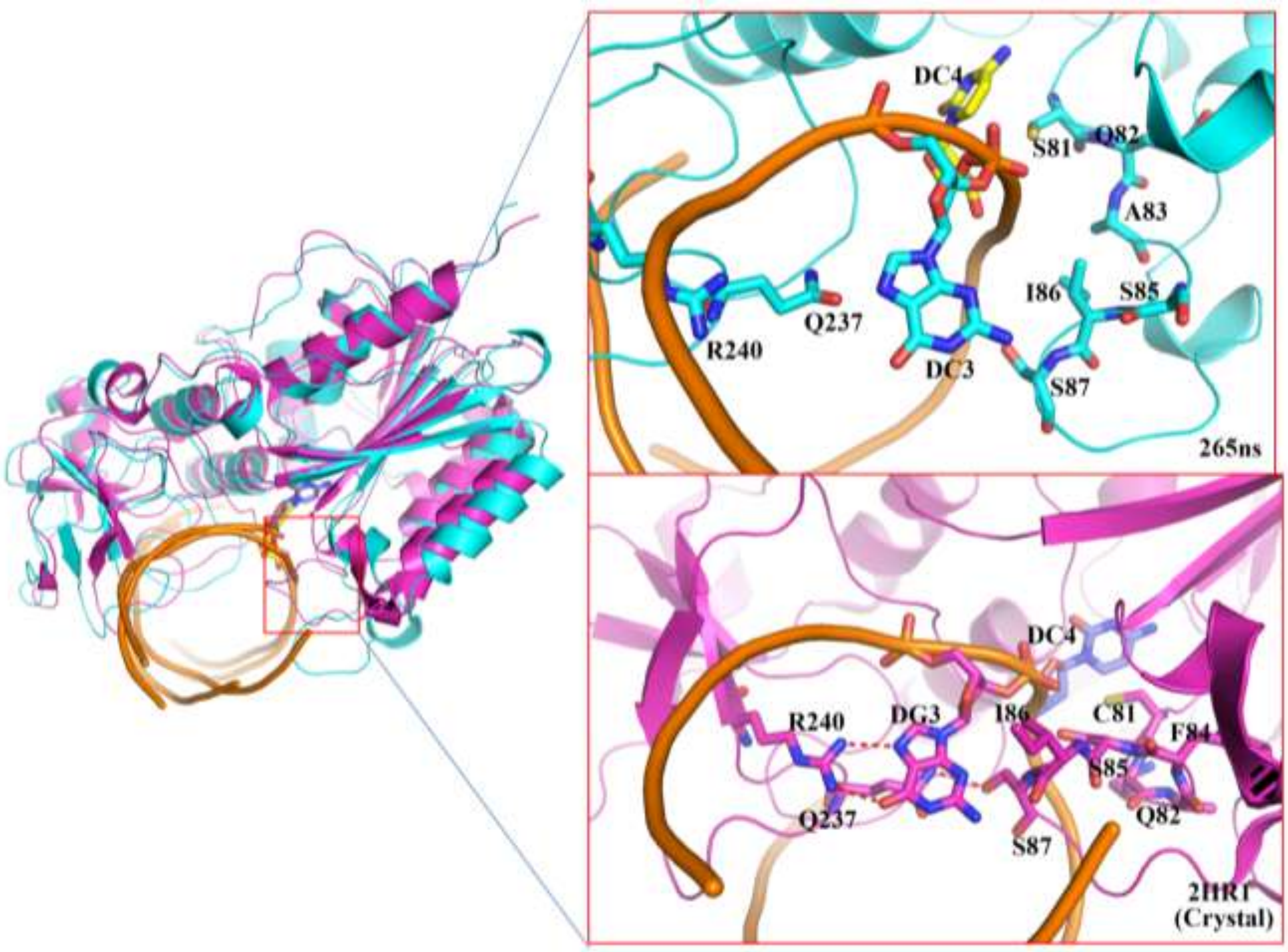


**Figure S2. A Comparison between the 265ns snapshot (the ending of our simulation) and the crystal structure of ternary complex.** Structure derived from simulation is colored cyan, while the

crystal structure is colored magenta. The residues that located around catalytic pocket are drawn using stick.

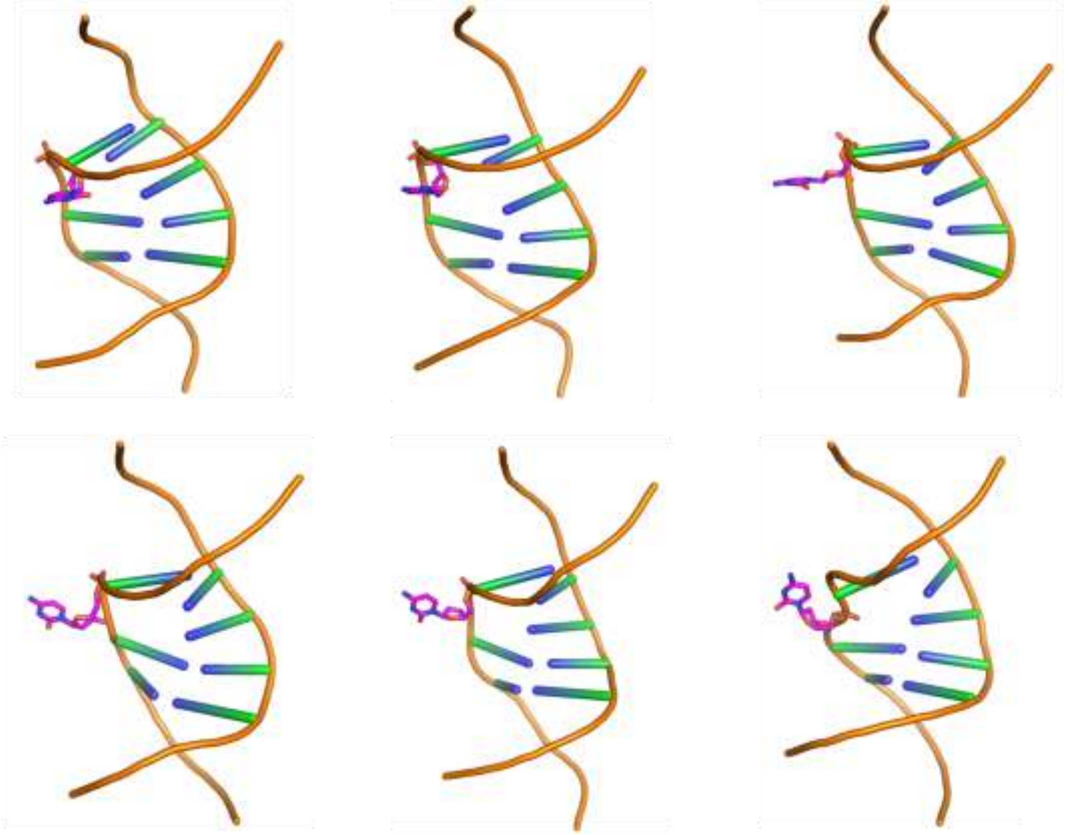

**Figure S3. Flipped base in semi-closed form.** Carbon atoms of target cytosine are colored magentas, while carbons of other bases are colored green. Other atoms are colored using default settings.

## Reference


1. Cheng, X.; Kumar, S.; Posfai, J.; Pflugrath, J. W.; Roberts, R. J. Crystal structure of the HhaI DNA methyltransferase complexed with S-adenosyl-L-methionine. *Cell* **1993,** *74* (2), 299-307.

2. O'Gara, M.; Zhang, X.; Roberts, R. J.; Cheng, X. Structure of a binary complex of HhaI methyltransferase with S-adnosyl-l-methionine formed in the presence of a short non-specific DNA oligonucleotide. *J. Mol. Biol.* **1999,** *287* (2), 201-209.

3. Song, J.; Rechkoblit, O.; Bestor, T. H.; Patel, D. J. Structure of DNMT1-DNA Complex Reveals a Role for Autoinhibition in Maintenance DNA Methylation. *Science* **2011,** *331* (6020), 1036-1040.

4. Van Der Spoel, D.; Lindahl, E.; Hess, B.; Groenhof, G.; Mark, A. E.; Berendsen, H. J. GROMACS: fast, flexible, and free. *J Comput Chem* **2005,** *26* (16), 1701-18.

5. Lavery, R.; Moakher, M.; Maddocks, J. H.; Petkeviciute, D.; Zakrzewska, K. Conformational analysis of nucleic acids revisited: Curves+. *Nucleic Acids Res.* **2009,** *37* (17), 5917-5929.

6. Baker, N. A.; Sept, D.; Joseph, S.; Holst, M. J.; McCammon, J. A. Electrostatics of nanosystems: Application to microtubules and the ribosome. *Proc. Natl. Acad. Sci. U. S. A.* **2001,** *98* (18), 10037-10041.

7. Team, R. D. C. *R: A Language and Environment for Statistical Computing*. 2011.

8. Humphrey, W.; Dalke, A.; Schulten, K. VMD: Visual molecular dynamics. *J. Mol. Graph. Model.* **1996,** *14* (1), 33-38.

9.Limongelli, V.; Bonomi, M.; Marinelli, L.; Gervasio, F. L.; Cavalli, A.; Novellino, E.; Parrinello, M. Molecular basis of cyclooxygenase enzymes (COXs) selective inhibition. *Proc. Natl. Acad. Sci. U. S. A.* **2010,** *107* (12), 5411-5416.

10. Huang, N.; Banavali, N. K.; MacKerell, A. D., Jr. Protein-facilitated base flipping in DNA by cytosine-5-methyltransferase. *Proc. Natl. Acad. Sci. U. S. A.* **2003,** *100* (1), 68-73.

11. Lacy, E. R.; Cox, K. K.; Wilson, W. D.; Lee, M. Recognition of T·G mismatched base pairs in DNA by stacked imidazole-containing polyamides: surface plasmon resonance and circular dichroism studies. *Nucleic Acids Res.* **2002,** *30* (8), 1834-1841.

12. Wang, H. H.; Xu, G.; Vonner, A. J.; Church, G. Modified bases enable high-efficiency oligonucleotide-mediated allelic replacement via mismatch repair evasion. *Nucleic Acids Res.* **2011,** *39* (16), 7336-7347.
